# Dehydro­abietic acid

**DOI:** 10.1107/S1600536809035600

**Published:** 2009-09-09

**Authors:** Xiao-Ping Rao, Zhan-Qian Song, Shi-Bin Shang

**Affiliations:** a Institute of Chemical Industry of Forest Products, Chinese Academy of Forestry, Nanjing 210042, People’s Republic of China

## Abstract

The title compound [systematic name: (1*R*,4a*S*,10a*R*)-7-iso­prop­yl-1,4a-dimethyl-1,2,3,4,4a,9,10,10a-octa­hydro­phen­anthrene-1-carboxylic acid], C_20_H_28_O_2_, has been isolated from disproportionated rosin which is obtained by isomerizing gum rosin with a Pd-C catalyst.. Two crystallographically independent mol­ecules exist in the asymmetric unit. In each mol­ecule, there are three six-membered rings, which adopt planar, half-chair and chair conformations. The two cyclo­hexane rings form a *trans* ring junction with the two methyl groups in axial positions. The crystal structure is stabilized by inter­molecular O—H⋯O hydrogen bonds.

## Related literature

For the synthesis and uses of dehydro­abietic acid, see: Halbrook & Lawrence (1966 ▶); Jia *et al.* (2009 ▶); Piispanen *et al.* (2001 ▶); Rao *et al.* (2006 ▶); Rao, Song & He (2008 ▶); Rao, Song, He & Jia (2008 ▶); Sepulveda *et al.* (2005 ▶); Wada *et al.* (1985 ▶). 
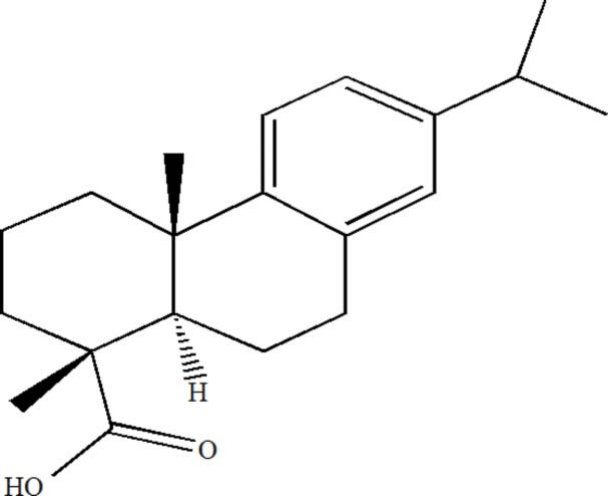


## Experimental

### 

#### Crystal data

C_20_H_28_O_2_
*M* *_r_* = 300.42
Monoclinic, 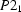

*a* = 11.738 (2) Å
*b* = 11.875 (2) Å
*c* = 13.654 (3) Å
β = 107.50 (3)°
*V* = 1815.1 (6) Å^3^
*Z* = 4
Mo *K*α radiation
μ = 0.07 mm^−1^
*T* = 293 K
0.40 × 0.20 × 0.20 mm

#### Data collection

Enraf–Nonius CAD-4 diffractometer
Absorption correction: ψ scan (*XCAD4*; Harms & Wocadlo, 1995 ▶) *T*_min_ = 0.973, *T*_max_ = 0.986
3592 measured reflections
3417 independent reflections
2173 reflections with *I* > 2σ(*I*)
*R*_int_ = 0.086
3 standard reflections every 200 reflections intensity decay: 1%

#### Refinement

*R*[*F* ^2^ > 2σ(*F* ^2^)] = 0.077
*wR*(*F* ^2^) = 0.182
*S* = 1.00
3417 reflections
361 parameters
3 restraints
H-atom parameters constrained
Δρ_max_ = 0.24 e Å^−3^
Δρ_min_ = −0.52 e Å^−3^

### 

Data collection: *CAD-4 Software* (Enraf–Nonius, 1985 ▶); cell refinement: *CAD-4 Software*; data reduction: *XCAD4* (Harms & Wocadlo, 1995 ▶); program(s) used to solve structure: *SHELXS97* (Sheldrick, 2008 ▶); program(s) used to refine structure: *SHELXL97* (Sheldrick, 2008 ▶); molecular graphics: *SHELXTL* (Sheldrick, 2008 ▶); software used to prepare material for publication: *SHELXTL*.

## Supplementary Material

Crystal structure: contains datablocks I, global. DOI: 10.1107/S1600536809035600/at2868sup1.cif

Structure factors: contains datablocks I. DOI: 10.1107/S1600536809035600/at2868Isup2.hkl

Additional supplementary materials:  crystallographic information; 3D view; checkCIF report

## Figures and Tables

**Table 1:** Hydrogen-bond geometry (Å, °)

*D*—H⋯*A*	*D*—H	H⋯*A*	*D*⋯*A*	*D*—H⋯*A*
O2—H2*D*⋯O3^i^	0.82	1.82	2.621 (8)	165
O4—H4*A*⋯O1^ii^	0.82	1.79	2.598 (8)	168

Symmetry codes: (i) 
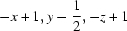
; (ii) 
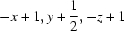
.

**Fig. 1.**
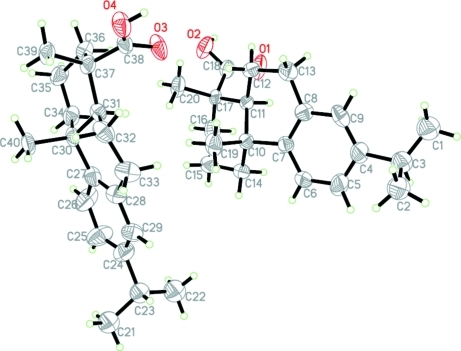
The molecular structure of the title compound, showing the atom labelling scheme. H atoms are represented by spheres of arbitrary radius and displacement ellipsoids are drawn at the 30% probability level.

## supplementary crystallographic information

### Comment

Dehydroabietic acid is an abietane diterpenic resin acid which can be easily obtained from Pinus resin or commercial disproportionated rosin (Halbrook & Lawrence, 1966). The tri-cyclic hydrophenanthrene structure of dehydroabietic acid has strong hydrophobicity, so it can be used as raw material for the synthesis of surfactants (Piispanen *et al.*, 2001; Jia *et al.*, 2009). Dehydroabietic acid is also widely used as starting material for design and synthesis of biological compounds (Sepulveda *et al.*, 2005; Rao, Song & He, 2008; Rao, Song He & Jia, 2008; Wada *et al.*, 1985). In this work, we describe the crystal structure of the title compound.

The overall geometry of the title compound (Fig. 1) is comparable to that found for dehydroabietic *N*-methyl anilide (Rao *et al., *2006) Two crystallorgraphic independent molecules exist in the asymmetric unit. In each molecule there are three six-membered rings, in which they form plannar, half-chair and chair conformations, respectively. The tricyclo phenanthrene structure of the title compound exhibited the same conformation with dehydroabietic *N*-methyl anilide. The two cyclohexane rings form a *trans* ring junction with two methyl groups in the same side of tricyclo phenanthrene structure. There are three chiral centers in each molecule, they exhibited R–, S– and R– configurations, respectively.

The crystal structure is stabilized by intermolecular O—H···O hydrogen bonds.

### Experimental

The title compound wasere isolated from disproportionated rosin by recrystallization 5 times from acetone. Single crystals were grown from acetone.

### Refinement

H atoms were positioned geometrically and refined as riding atoms, with C—H = 0.96Å and *U*_iso_(H) = 1.5*U*_eq_(C) for methyl H atoms, and C—H = 0.97–0.98Å and *U*_iso_(H) = 1.2*U*_eq_(C) for all other H atoms.

### Crystal data

**Table:**

C_20_H_28_O_2_	*F*(000) = 656
*M**_r_* = 300.42	*D*_x_ = 1.099 Mg m^−^^3^
Monoclinic, *P*2_1_	Mo *K*α radiation, λ = 0.71073 Å
Hall symbol: P 2yb	Cell parameters from 25 reflections
*a* = 11.738 (2) Å	θ = 9–13°
*b* = 11.875 (2) Å	µ = 0.07 mm^−^^1^
*c* = 13.654 (3) Å	*T* = 293 K
β = 107.50 (3)°	Block, white
*V* = 1815.1 (6) Å^3^	0.40 × 0.20 × 0.20 mm
*Z* = 4	

### Data collection

**Table:**

Enraf–Nonius CAD-4 diffractometer	2173 reflections with *I* > 2σ(*I*)
Radiation source: fine-focus sealed tube	*R*_int_ = 0.086
graphite	θ_max_ = 25.2°, θ_min_ = 1.6°
ω/2θ scans	*h* = 0→14
Absorption correction: ψ scan (*XCAD4*; Harms & Wocadlo, 1995)	*k* = 0→14
*T*_min_ = 0.973, *T*_max_ = 0.986	*l* = −16→15
3592 measured reflections	3 standard reflections every 200 reflections
3417 independent reflections	intensity decay: 1%

### Refinement

**Table:**

Refinement on *F*^2^	Primary atom site location: structure-invariant direct methods
Least-squares matrix: full	Secondary atom site location: difference Fourier map
*R*[*F*^2^ > 2σ(*F*^2^)] = 0.077	Hydrogen site location: inferred from neighbouring sites
*wR*(*F*^2^) = 0.182	H-atom parameters constrained
*S* = 1.00	*w* = 1/[σ^2^(*F*_o_^2^) + (0.06*P*)^2^ + 1.6*P*] where *P* = (*F*_o_^2^ + 2*F*_c_^2^)/3
3417 reflections	(Δ/σ)_max_ < 0.001
361 parameters	Δρ_max_ = 0.24 e Å^−^^3^
3 restraints	Δρ_min_ = −0.52 e Å^−^^3^

### Special details

**Table:**

Geometry. All e.s.d.'s (except the e.s.d. in the dihedral angle between two l.s. planes) are estimated using the full covariance matrix. The cell e.s.d.'s are taken into account individually in the estimation of e.s.d.'s in distances, angles and torsion angles; correlations between e.s.d.'s in cell parameters are only used when they are defined by crystal symmetry. An approximate (isotropic) treatment of cell e.s.d.'s is used for estimating e.s.d.'s involving l.s. planes.
Refinement. Refinement of *F*^2^ against ALL reflections. The weighted *R*-factor *wR* and goodness of fit *S* are based on *F*^2^, conventional *R*-factors *R* are based on *F*, with *F* set to zero for negative *F*^2^. The threshold expression of *F*^2^ > σ(*F*^2^) is used only for calculating *R*-factors(gt) *etc*. and is not relevant to the choice of reflections for refinement. *R*-factors based on *F*^2^ are statistically about twice as large as those based on *F*, and *R*- factors based on ALL data will be even larger.

### Fractional atomic coordinates and isotropic or equivalent isotropic displacement parameters (Å^2^)

**Table:**

	*x*	*y*	*z*	*U*_iso_*/*U*_eq_	
O1	0.7903 (4)	0.0770 (5)	0.5980 (5)	0.111 (2)	
O2	0.6194 (4)	−0.0132 (5)	0.5509 (4)	0.0981 (18)	
H2D	0.6584	−0.0487	0.5206	0.147*	
C1	1.0216 (7)	0.7319 (9)	0.7227 (6)	0.105	
H1A	1.0473	0.6592	0.7068	0.158*	
H1B	1.0879	0.7832	0.7385	0.158*	
H1C	0.9594	0.7594	0.6646	0.158*	
C2	0.9229 (7)	0.8140 (7)	0.8668 (6)	0.094	
H2A	0.8972	0.7796	0.9202	0.141*	
H2B	0.8559	0.8493	0.8180	0.141*	
H2C	0.9827	0.8697	0.8961	0.141*	
C3	0.9762 (7)	0.7223 (7)	0.8115 (5)	0.089	
H3A	1.0471	0.6976	0.8660	0.107*	
C4	0.8968 (8)	0.6219 (7)	0.7975 (7)	0.094 (2)	
C5	0.9072 (8)	0.5624 (8)	0.8875 (7)	0.104 (3)	
H5A	0.9543	0.5913	0.9499	0.125*	
C6	0.8482 (6)	0.4603 (7)	0.8856 (5)	0.079 (2)	
H6A	0.8596	0.4206	0.9465	0.095*	
C7	0.7739 (5)	0.4168 (5)	0.7964 (4)	0.0580 (15)	
C8	0.7635 (6)	0.4722 (6)	0.7055 (5)	0.0660 (16)	
C9	0.8238 (7)	0.5731 (6)	0.7076 (6)	0.085 (2)	
H9A	0.8151	0.6101	0.6457	0.102*	
C10	0.7026 (5)	0.3060 (5)	0.7991 (4)	0.0554 (15)	
C11	0.6944 (4)	0.2442 (5)	0.6962 (4)	0.0463 (13)	
H11A	0.7771	0.2384	0.6946	0.056*	
C12	0.6319 (5)	0.3137 (6)	0.6046 (4)	0.0636 (16)	
H12A	0.5501	0.3259	0.6042	0.076*	
H12B	0.6299	0.2722	0.5429	0.076*	
C13	0.6904 (7)	0.4267 (7)	0.6020 (5)	0.085 (2)	
H13A	0.6285	0.4810	0.5704	0.101*	
H13B	0.7419	0.4203	0.5585	0.101*	
C14	0.7656 (6)	0.2326 (6)	0.8889 (4)	0.0674 (17)	
H14A	0.7627	0.2688	0.9518	0.081*	
H14B	0.8489	0.2255	0.8917	0.081*	
C15	0.7105 (7)	0.1152 (6)	0.8826 (5)	0.084 (2)	
H15A	0.7548	0.0714	0.9418	0.101*	
H15B	0.6288	0.1217	0.8844	0.101*	
C16	0.7117 (6)	0.0560 (6)	0.7883 (6)	0.081 (2)	
H16A	0.6743	−0.0170	0.7869	0.097*	
H16B	0.7940	0.0434	0.7903	0.097*	
C17	0.6479 (4)	0.1187 (6)	0.6895 (4)	0.0549 (15)	
C19	0.5766 (5)	0.3435 (7)	0.8062 (6)	0.085 (2)	
H19A	0.5293	0.2780	0.8080	0.128*	
H19B	0.5369	0.3884	0.7473	0.128*	
H19C	0.5867	0.3869	0.8675	0.128*	
C18	0.6865 (5)	0.0604 (5)	0.6094 (5)	0.0623 (16)	
C20	0.5112 (5)	0.1113 (6)	0.6643 (6)	0.082 (2)	
H20A	0.4874	0.0336	0.6609	0.123*	
H20B	0.4741	0.1468	0.5994	0.123*	
H20C	0.4866	0.1487	0.7169	0.123*	
O3	0.2917 (4)	0.3532 (5)	0.5604 (4)	0.0990 (19)	
O4	0.1213 (4)	0.4452 (5)	0.5126 (4)	0.0960 (18)	
H4A	0.1581	0.4809	0.4806	0.144*	
C21	0.4859 (7)	0.2433 (8)	1.2965 (6)	0.098	
H21A	0.5408	0.2628	1.3620	0.148*	
H21B	0.4750	0.1631	1.2926	0.148*	
H21C	0.4105	0.2793	1.2887	0.148*	
C22	0.6600 (6)	0.2601 (9)	1.2160 (6)	0.107	
H22A	0.7119	0.2770	1.2834	0.160*	
H22B	0.6806	0.3068	1.1664	0.160*	
H22C	0.6689	0.1824	1.2004	0.160*	
C23	0.5353 (6)	0.2817 (7)	1.2122 (5)	0.085	
H23A	0.5325	0.3640	1.2170	0.102*	
C24	0.4502 (6)	0.2576 (9)	1.1044 (6)	0.093 (2)	
C25	0.4036 (8)	0.1542 (8)	1.0763 (7)	0.112 (3)	
H25A	0.4219	0.0958	1.1239	0.134*	
C26	0.3299 (7)	0.1337 (7)	0.9789 (7)	0.108 (3)	
H26A	0.3076	0.0599	0.9597	0.129*	
C27	0.2870 (5)	0.2205 (6)	0.9074 (5)	0.0695 (19)	
C28	0.3299 (6)	0.3285 (6)	0.9408 (5)	0.0709 (18)	
C29	0.4126 (6)	0.3443 (6)	1.0387 (5)	0.0747 (19)	
H29A	0.4419	0.4162	1.0586	0.090*	
C30	0.1924 (5)	0.1988 (6)	0.8056 (5)	0.0650 (18)	
C31	0.2050 (5)	0.2939 (5)	0.7340 (4)	0.0540 (14)	
H31A	0.2888	0.2904	0.7352	0.065*	
C32	0.1918 (7)	0.4097 (6)	0.7754 (5)	0.079 (2)	
H32A	0.1163	0.4150	0.7905	0.095*	
H32B	0.1927	0.4665	0.7246	0.095*	
C33	0.2942 (7)	0.4292 (6)	0.8722 (5)	0.084 (2)	
H33A	0.3630	0.4545	0.8529	0.101*	
H33B	0.2722	0.4894	0.9110	0.101*	
C34	0.2114 (6)	0.0853 (6)	0.7582 (5)	0.0736 (19)	
H34A	0.2947	0.0796	0.7609	0.088*	
H34B	0.1953	0.0252	0.8004	0.088*	
C35	0.1367 (6)	0.0670 (7)	0.6509 (5)	0.087 (2)	
H35A	0.0534	0.0631	0.6486	0.105*	
H35B	0.1580	−0.0043	0.6266	0.105*	
C36	0.1528 (6)	0.1607 (7)	0.5810 (5)	0.078 (2)	
H36A	0.0986	0.1477	0.5127	0.094*	
H36B	0.2336	0.1571	0.5763	0.094*	
C37	0.1309 (5)	0.2781 (6)	0.6151 (5)	0.0650 (17)	
C39	−0.0057 (5)	0.3022 (8)	0.5926 (5)	0.087 (2)	
H39A	−0.0457	0.2913	0.5208	0.130*	
H39B	−0.0169	0.3784	0.6112	0.130*	
H39C	−0.0384	0.2516	0.6320	0.130*	
C38	0.1848 (5)	0.3630 (6)	0.5582 (5)	0.0688 (19)	
C40	0.0688 (5)	0.1971 (8)	0.8262 (5)	0.090 (3)	
H40A	0.0568	0.2672	0.8567	0.136*	
H40B	0.0668	0.1363	0.8719	0.136*	
H40C	0.0066	0.1868	0.7625	0.136*	

### Atomic displacement parameters (Å^2^)

**Table:**

	*U*^11^	*U*^22^	*U*^33^	*U*^12^	*U*^13^	*U*^23^
O1	0.076 (3)	0.120 (5)	0.161 (5)	−0.044 (3)	0.070 (3)	−0.075 (4)
O2	0.058 (3)	0.116 (4)	0.124 (4)	−0.022 (3)	0.032 (3)	−0.060 (4)
C1	0.105	0.105	0.105	0.000	0.032	0.000
C2	0.094	0.094	0.094	0.000	0.028	0.000
C3	0.089	0.089	0.089	0.000	0.027	0.000
C4	0.131 (7)	0.069 (5)	0.094 (6)	−0.045 (5)	0.052 (5)	−0.031 (5)
C5	0.127 (7)	0.088 (6)	0.113 (7)	−0.054 (5)	0.060 (5)	−0.055 (6)
C6	0.094 (5)	0.094 (6)	0.060 (4)	−0.027 (4)	0.037 (4)	−0.015 (4)
C7	0.066 (4)	0.055 (4)	0.057 (4)	−0.007 (3)	0.026 (3)	−0.008 (3)
C8	0.073 (4)	0.057 (4)	0.065 (4)	0.003 (3)	0.015 (3)	0.005 (3)
C9	0.105 (5)	0.070 (5)	0.092 (5)	−0.007 (4)	0.051 (4)	0.022 (4)
C10	0.048 (3)	0.065 (4)	0.059 (4)	0.000 (3)	0.024 (3)	−0.008 (3)
C11	0.040 (3)	0.056 (3)	0.046 (3)	0.002 (3)	0.017 (2)	−0.009 (3)
C12	0.065 (3)	0.068 (4)	0.053 (3)	−0.008 (3)	0.010 (3)	−0.006 (3)
C13	0.084 (5)	0.091 (5)	0.075 (5)	−0.004 (4)	0.019 (4)	0.020 (4)
C14	0.076 (4)	0.075 (4)	0.052 (3)	−0.009 (4)	0.020 (3)	0.000 (3)
C15	0.105 (5)	0.079 (5)	0.070 (4)	−0.018 (4)	0.027 (4)	0.005 (4)
C16	0.081 (5)	0.058 (4)	0.108 (6)	−0.008 (4)	0.036 (4)	0.005 (4)
C17	0.031 (2)	0.079 (4)	0.058 (3)	−0.006 (3)	0.018 (2)	−0.009 (3)
C19	0.068 (4)	0.089 (5)	0.115 (6)	−0.003 (4)	0.053 (4)	−0.025 (5)
C18	0.050 (3)	0.057 (4)	0.083 (4)	0.001 (3)	0.025 (3)	−0.008 (4)
C20	0.056 (3)	0.086 (5)	0.114 (6)	−0.020 (4)	0.041 (4)	−0.036 (5)
O3	0.052 (2)	0.135 (5)	0.122 (4)	0.028 (3)	0.043 (3)	0.071 (4)
O4	0.062 (3)	0.112 (4)	0.117 (4)	0.016 (3)	0.032 (3)	0.066 (4)
C21	0.098	0.098	0.098	0.000	0.030	0.000
C22	0.107	0.107	0.107	0.000	0.032	0.000
C23	0.085	0.085	0.085	0.000	0.026	0.000
C24	0.081 (5)	0.107 (7)	0.078 (5)	−0.006 (5)	0.006 (4)	0.001 (5)
C25	0.139 (8)	0.084 (6)	0.081 (5)	−0.014 (6)	−0.015 (5)	0.021 (5)
C26	0.102 (6)	0.074 (5)	0.113 (7)	−0.022 (5)	−0.017 (5)	0.029 (5)
C27	0.058 (4)	0.076 (5)	0.076 (4)	0.010 (3)	0.023 (3)	0.031 (4)
C28	0.076 (4)	0.072 (5)	0.067 (4)	0.016 (4)	0.024 (3)	0.002 (4)
C29	0.091 (5)	0.069 (5)	0.067 (4)	0.008 (4)	0.028 (4)	−0.009 (4)
C30	0.040 (3)	0.088 (5)	0.073 (4)	0.002 (3)	0.025 (3)	0.017 (4)
C31	0.049 (3)	0.058 (4)	0.060 (3)	0.014 (3)	0.023 (3)	0.010 (3)
C32	0.089 (5)	0.077 (5)	0.084 (5)	0.020 (4)	0.043 (4)	0.018 (4)
C33	0.122 (6)	0.055 (4)	0.076 (5)	0.017 (4)	0.031 (4)	0.000 (4)
C34	0.066 (4)	0.061 (4)	0.090 (5)	−0.011 (3)	0.018 (4)	0.011 (4)
C35	0.079 (5)	0.083 (5)	0.087 (5)	−0.027 (4)	0.005 (4)	0.013 (4)
C36	0.059 (4)	0.094 (6)	0.077 (4)	−0.011 (4)	0.015 (3)	−0.005 (4)
C37	0.038 (3)	0.084 (5)	0.070 (4)	0.005 (3)	0.011 (3)	0.019 (4)
C39	0.039 (3)	0.125 (6)	0.097 (5)	0.010 (4)	0.020 (3)	0.032 (5)
C38	0.042 (3)	0.099 (5)	0.067 (4)	0.007 (3)	0.019 (3)	0.029 (4)
C40	0.052 (3)	0.138 (7)	0.088 (4)	−0.011 (4)	0.032 (3)	0.042 (5)

### Geometric parameters (Å, °)

**Table:**

O1—C18	1.289 (7)	O3—C38	1.252 (6)
O2—C18	1.281 (7)	O4—C38	1.271 (8)
O2—H2D	0.8200	O4—H4A	0.8200
C1—C3	1.469 (7)	C21—C23	1.507 (10)
C1—H1A	0.9600	C21—H21A	0.9600
C1—H1B	0.9600	C21—H21B	0.9600
C1—H1C	0.9600	C21—H21C	0.9600
C2—C3	1.559 (10)	C22—C23	1.472 (7)
C2—H2A	0.9600	C22—H22A	0.9600
C2—H2B	0.9600	C22—H22B	0.9600
C2—H2C	0.9600	C22—H22C	0.9600
C3—C4	1.490 (10)	C23—C24	1.538 (10)
C3—H3A	0.9800	C23—H23A	0.9800
C4—C5	1.391 (12)	C24—C29	1.350 (10)
C4—C9	1.395 (10)	C24—C25	1.352 (12)
C5—C6	1.393 (10)	C25—C26	1.373 (11)
C5—H5A	0.9300	C25—H25A	0.9300
C6—C7	1.368 (8)	C26—C27	1.405 (9)
C6—H6A	0.9300	C26—H26A	0.9300
C7—C8	1.377 (8)	C27—C28	1.404 (10)
C7—C10	1.567 (9)	C27—C30	1.518 (9)
C8—C9	1.388 (9)	C28—C29	1.408 (9)
C8—C13	1.516 (9)	C28—C33	1.498 (10)
C9—H9A	0.9300	C29—H29A	0.9300
C10—C14	1.504 (9)	C30—C31	1.530 (8)
C10—C11	1.562 (7)	C30—C34	1.540 (10)
C10—C19	1.576 (7)	C30—C40	1.558 (7)
C11—C12	1.494 (8)	C31—C32	1.513 (9)
C11—C17	1.581 (8)	C31—C37	1.605 (8)
C11—H11A	0.9800	C31—H31A	0.9800
C12—C13	1.511 (10)	C32—C33	1.514 (10)
C12—H12A	0.9700	C32—H32A	0.9700
C12—H12B	0.9700	C32—H32B	0.9700
C13—H13A	0.9700	C33—H33A	0.9700
C13—H13B	0.9700	C33—H33B	0.9700
C14—C15	1.529 (10)	C34—C35	1.480 (9)
C14—H14A	0.9700	C34—H34A	0.9700
C14—H14B	0.9700	C34—H34B	0.9700
C15—C16	1.471 (10)	C35—C36	1.515 (10)
C15—H15A	0.9700	C35—H35A	0.9700
C15—H15B	0.9700	C35—H35B	0.9700
C16—C17	1.524 (9)	C36—C37	1.516 (10)
C16—H16A	0.9700	C36—H36A	0.9700
C16—H16B	0.9700	C36—H36B	0.9700
C17—C18	1.477 (8)	C37—C38	1.522 (9)
C17—C20	1.540 (7)	C37—C39	1.566 (7)
C19—H19A	0.9600	C39—H39A	0.9600
C19—H19B	0.9600	C39—H39B	0.9600
C19—H19C	0.9600	C39—H39C	0.9600
C20—H20A	0.9600	C40—H40A	0.9600
C20—H20B	0.9600	C40—H40B	0.9600
C20—H20C	0.9600	C40—H40C	0.9600
			
C18—O2—H2D	109.5	C38—O4—H4A	109.5
C3—C1—H1A	109.5	C23—C21—H21A	109.5
C3—C1—H1B	109.5	C23—C21—H21B	109.5
H1A—C1—H1B	109.5	H21A—C21—H21B	109.5
C3—C1—H1C	109.5	C23—C21—H21C	109.5
H1A—C1—H1C	109.5	H21A—C21—H21C	109.5
H1B—C1—H1C	109.5	H21B—C21—H21C	109.5
C3—C2—H2A	109.5	C23—C22—H22A	109.5
C3—C2—H2B	109.5	C23—C22—H22B	109.5
H2A—C2—H2B	109.5	H22A—C22—H22B	109.5
C3—C2—H2C	109.5	C23—C22—H22C	109.5
H2A—C2—H2C	109.5	H22A—C22—H22C	109.5
H2B—C2—H2C	109.5	H22B—C22—H22C	109.5
C1—C3—C4	108.9 (7)	C22—C23—C21	121.9 (7)
C1—C3—C2	130.1 (7)	C22—C23—C24	110.7 (6)
C4—C3—C2	107.1 (6)	C21—C23—C24	112.7 (6)
C1—C3—H3A	102.4	C22—C23—H23A	102.9
C4—C3—H3A	102.4	C21—C23—H23A	102.9
C2—C3—H3A	102.4	C24—C23—H23A	102.9
C5—C4—C9	115.8 (7)	C29—C24—C25	118.7 (7)
C5—C4—C3	114.1 (7)	C29—C24—C23	118.8 (8)
C9—C4—C3	129.8 (7)	C25—C24—C23	122.1 (8)
C4—C5—C6	121.0 (7)	C24—C25—C26	121.3 (8)
C4—C5—H5A	119.5	C24—C25—H25A	119.4
C6—C5—H5A	119.5	C26—C25—H25A	119.4
C7—C6—C5	121.7 (7)	C25—C26—C27	122.3 (8)
C7—C6—H6A	119.2	C25—C26—H26A	118.9
C5—C6—H6A	119.2	C27—C26—H26A	118.9
C6—C7—C8	118.9 (6)	C28—C27—C26	115.2 (6)
C6—C7—C10	119.9 (6)	C28—C27—C30	123.0 (6)
C8—C7—C10	121.3 (5)	C26—C27—C30	121.5 (7)
C7—C8—C9	119.2 (6)	C27—C28—C29	120.5 (6)
C7—C8—C13	122.8 (6)	C27—C28—C33	121.4 (6)
C9—C8—C13	118.0 (6)	C29—C28—C33	118.0 (7)
C8—C9—C4	123.4 (7)	C24—C29—C28	121.6 (7)
C8—C9—H9A	118.3	C24—C29—H29A	119.2
C4—C9—H9A	118.3	C28—C29—H29A	119.2
C14—C10—C11	110.1 (5)	C27—C30—C31	105.9 (5)
C14—C10—C7	111.7 (5)	C27—C30—C34	112.0 (5)
C11—C10—C7	105.2 (4)	C31—C30—C34	108.9 (5)
C14—C10—C19	110.7 (5)	C27—C30—C40	107.6 (5)
C11—C10—C19	112.5 (5)	C31—C30—C40	113.5 (5)
C7—C10—C19	106.4 (5)	C34—C30—C40	108.9 (6)
C12—C11—C10	112.0 (5)	C32—C31—C30	113.1 (5)
C12—C11—C17	113.5 (4)	C32—C31—C37	113.5 (5)
C10—C11—C17	114.9 (4)	C30—C31—C37	115.6 (5)
C12—C11—H11A	105.1	C32—C31—H31A	104.4
C10—C11—H11A	105.1	C30—C31—H31A	104.4
C17—C11—H11A	105.1	C37—C31—H31A	104.4
C11—C12—C13	113.5 (5)	C31—C32—C33	108.8 (6)
C11—C12—H12A	108.9	C31—C32—H32A	109.9
C13—C12—H12A	108.9	C33—C32—H32A	109.9
C11—C12—H12B	108.9	C31—C32—H32B	109.9
C13—C12—H12B	108.9	C33—C32—H32B	109.9
H12A—C12—H12B	107.7	H32A—C32—H32B	108.3
C12—C13—C8	115.4 (6)	C28—C33—C32	115.0 (7)
C12—C13—H13A	108.4	C28—C33—H33A	108.5
C8—C13—H13A	108.4	C32—C33—H33A	108.5
C12—C13—H13B	108.4	C28—C33—H33B	108.5
C8—C13—H13B	108.4	C32—C33—H33B	108.5
H13A—C13—H13B	107.5	H33A—C33—H33B	107.5
C10—C14—C15	112.8 (5)	C35—C34—C30	115.2 (6)
C10—C14—H14A	109.0	C35—C34—H34A	108.5
C15—C14—H14A	109.0	C30—C34—H34A	108.5
C10—C14—H14B	109.0	C35—C34—H34B	108.5
C15—C14—H14B	109.0	C30—C34—H34B	108.5
H14A—C14—H14B	107.8	H34A—C34—H34B	107.5
C16—C15—C14	111.7 (6)	C34—C35—C36	111.6 (6)
C16—C15—H15A	109.3	C34—C35—H35A	109.3
C14—C15—H15A	109.3	C36—C35—H35A	109.3
C16—C15—H15B	109.3	C34—C35—H35B	109.3
C14—C15—H15B	109.3	C36—C35—H35B	109.3
H15A—C15—H15B	107.9	H35A—C35—H35B	108.0
C15—C16—C17	114.2 (6)	C35—C36—C37	114.6 (6)
C15—C16—H16A	108.7	C35—C36—H36A	108.6
C17—C16—H16A	108.7	C37—C36—H36A	108.6
C15—C16—H16B	108.7	C35—C36—H36B	108.6
C17—C16—H16B	108.7	C37—C36—H36B	108.6
H16A—C16—H16B	107.6	H36A—C36—H36B	107.6
C18—C17—C16	104.4 (5)	C36—C37—C38	108.5 (5)
C18—C17—C20	109.8 (5)	C36—C37—C39	111.5 (6)
C16—C17—C20	111.8 (5)	C38—C37—C39	109.7 (5)
C18—C17—C11	107.9 (4)	C36—C37—C31	109.2 (5)
C16—C17—C11	109.9 (5)	C38—C37—C31	104.9 (5)
C20—C17—C11	112.7 (5)	C39—C37—C31	112.9 (5)
C10—C19—H19A	109.5	C37—C39—H39A	109.5
C10—C19—H19B	109.5	C37—C39—H39B	109.5
H19A—C19—H19B	109.5	H39A—C39—H39B	109.5
C10—C19—H19C	109.5	C37—C39—H39C	109.5
H19A—C19—H19C	109.5	H39A—C39—H39C	109.5
H19B—C19—H19C	109.5	H39B—C39—H39C	109.5
O2—C18—O1	117.1 (6)	O3—C38—O4	121.3 (6)
O2—C18—C17	120.9 (5)	O3—C38—C37	119.6 (6)
O1—C18—C17	121.9 (6)	O4—C38—C37	119.1 (5)
C17—C20—H20A	109.5	C30—C40—H40A	109.5
C17—C20—H20B	109.5	C30—C40—H40B	109.5
H20A—C20—H20B	109.5	H40A—C40—H40B	109.5
C17—C20—H20C	109.5	C30—C40—H40C	109.5
H20A—C20—H20C	109.5	H40A—C40—H40C	109.5
H20B—C20—H20C	109.5	H40B—C40—H40C	109.5

### Hydrogen-bond geometry (Å, °)

**Table:**

*D*—H···*A*	*D*—H	H···*A*	*D*···*A*	*D*—H···*A*
O2—H2D···O3^i^	0.82	1.82	2.621 (8)	165
O4—H4A···O1^ii^	0.82	1.79	2.598 (8)	168
C11—H11A···O1	0.98	2.36	2.813 (8)	108
C31—H31A···O3	0.98	2.51	2.933 (8)	106
C36—H36B···O3	0.97	2.45	2.870 (10)	105

Symmetry codes: (i) −*x*+1, *y*−1/2, −*z*+1; (ii) −*x*+1, *y*+1/2, −*z*+1.
